# Case of Reduction of Dislocation of the Hip on the Dorsum Ilii, by Dr. Reid’s Method

**Published:** 1852-01

**Authors:** S. G. Bailey


ART. IV.—*Case of Reduction of Dislocation of the Hip on the Dorsum Ilii, by Dr. Reid's method.* S. G. BAILEY, M. D.

DR. FLINT:

*Dear Sir:*—The following case may be of interest, so far, at least, as relates to the recently suggested operation for reduction of dislocation of the femur on the dorsum ilii by your correspondent, Dr. Reid, of Rochester, N. Y., to whom, through your Journal, I think that both the profession and patients, in such cases, are much indebted.

Nov. 22d, 1851.—James Doyle, laborer, fell from a pile of coal, on the deck of a vessel, into the hold. The depth of the hold was about eight feet and the pile of coal on which he stood elevated him about two feet from the deck. He had lifted a large piece of coal to carry to a wheelbarrow, on deck, when a piece of the coal rolled under one of his feet, and precipitated him over the edge of the hatch-way to the bottom of the hold, across the keelson. He struck upon the right limb and side, and was lying in that position when taken up. I saw him in twenty minutes after the accident, in an office near the vessel. He was then lying on his back manifesting much pain, the right limb a little drawn up, resting over against the other, the heel turned out and the inside of the foot resting upon the floor. He complained principally of his hip. Said he was hurt most there, and before removing his pantaloons, I attempted to straighten and examine the limb, but desisted immediately on account of his shouts from pain. I now removed his pantaloons easily by holding the limb in the semi-flexed position that I found it, and the character of the injury was perceptible at a glance. I had read Dr. Reid's paper, in the Journal for August, carefully, and with much interest, upon the subject of dislocation of the femur on the *dorsum ilii.* Without any definite idea of what the result would be, and desirous of affording the patient as speedy relief as possible, I proceeded, at once, with the assistance of two men, to hold the patient firmly, to put the operation of Dr. R. in practice. In doing so, I need only say that I followed the directions, exactly, as given in the above named paper.

The reduction was easily accomplished, and gave but little pain, comparatively, to the patient, though he made some out-cry. The head of the bone returned to its place, with the usual report, which was observed by the bystanders, and the patient, with others, exclaimed, " that 's it!" The pain was relieved at that point, though he was still in pain from accompanying injuries. He has passed considerable bloody urine since, had much pain in his privates, through the pelvis and in the lower right limb. Directly after the reduction, he was conveyed to his house, and is now, Dec. 4th, able to move the limb quite freely, but does not yet attempt to step on it.

Having seen this injury but once before, I was desirous that some one of more experience should examine the patient, and am obliged to Dr. A. S. Sprague, of this city, for so doing, who saw the patient in about an hour after he was taken home. Dr. Hawley and a young gentleman from Dr. Sprague's office were also present with Dr. S.

The patient is now doing well and appearances indicate a rapid recovery.

Very Respectfully Yours, &c.

SAM'L G. BAILEY.

